# Obesity-depression-metabolism triad: amplification of cardiometabolic multimorbidity risk in Chinese adults (CHARLS 2011–2018)

**DOI:** 10.1186/s12944-025-02724-1

**Published:** 2025-09-29

**Authors:** Qinglin He, Wen Yu, Chubing Long, Dong Lin, Feng Lu, Maoling Zhong, Wei Lin, Shupeng Huang, Shuiying Hua, Fuzhou Hua, Xifeng Wang

**Affiliations:** 1 Department of Anesthesiology, the Second Affiliated Hospital, Jiangxi Medical College, Nanchang University, Minde Road, Nanchang, Jiangxi Province 330006 China; 2 Key Laboratory of Anesthesiology of Jiangxi Province, #1 Minde Road, Nanchang City, Jiangxi Province 330006 P. R. China; 3 Department of Anesthesiology, The First Affiliated Hospital, Jiangxi Medical College, Nanchang University, N17# Yong Wai Zheng Street, Nanchang, Jiangxi Province 330006 P. R. China; 4 Department of Anesthesiology, The First Affiliated Hospital of Gannan Medical University, No.128, Jinling Road, Zhanggong District, Ganzhou City, Jiangxi Province 34100 P. R. China

**Keywords:** TyG, BRI, Depression, Cardiometabolic multimorbidity, CHARLS

## Abstract

**Background:**

Previous studies have shown that obesity and metabolic and psychological factors are related to cardiometabolic comorbidities, and this study focuses on the BRI, TyG index and depression and explores the independent and combined effects of these three factors on cardiometabolic comorbidities from multiple perspectives.

**Methods:**

We analyzed data from 5,199 participants (baseline 2011) in the China Health and Retirement Longitudinal Study (CHARLS). The participants were stratified into four groups on the basis of the median TyG index (8.62) and BRI (4.03). Logistic regression models were used to assess associations between TyG-BRI strata, depression (CES-D score ≥ 10), and incident CMM (cardiometabolic multimorbidity), incorporating multiplicative interaction terms (BRI×TyG×Depression) and additive interaction indices (RERI/AP). A composite score was developed using standardized biomarker z scores and depression status, with predicti·ve performance evaluated via ROC-AUC comparisons (DeLong’s test) and restricted cubic splines.

**Results:**

During the 7-year follow-up period, 331 incident CMM cases were documented. Machine learning models (random forest and XGBoost) identified BRI and TyG as the top predictors of CMM. Logistic regression revealed the highest risk in the high-BRI & high-TyG group (OR = 3.89, 95% CI = 3.02–5.01 vs. low-BRI and low-TyG). Tripartite exposure (high BRI and high TyG and depression) was associated with a striking 14-fold increased risk (OR = 14.2, 95% CI = 7.95–27.6), with a significant multiplicative interaction (*p* = 0.016). The composite cardiometabolic‒psychosocial score showed superior predictive capacity (AUC = 0.70 vs. BRI = 0.66, TyG = 0.65, depression = 0.56; DeLong’s *p* < 0.001) and dose‒responsive risk escalation (P-overall < 0.001, P for nonlinearity = 0.70).

**Conclusion:**

This study identifies BRI, TyG, and depression as independent and synergistic predictors of CMM. Our findings advocate integrated clinical strategies for effective CMM prevention.

**Supplementary Information:**

The online version contains supplementary material available at 10.1186/s12944-025-02724-1.

## Introduction

Cardiometabolic multimorbidity (CMM), defined as the co-occurrence of cardiovascular diseases (e.g., coronary heart disease, stroke) and metabolic disorders (e.g., type 2 diabetes, obesity), has emerged as a critical global public health challenge [1, 2]. This complex clinical entity not only substantially elevates all-cause mortality and healthcare burdens but also presents intricate management dilemmas [1]. While traditional risk factors (e.g., hypertension and hyperglycemia) have been extensively investigated [3–5], the synergistic mechanisms involving novel biomarkers and psychosocial determinants remain incompletely understood.

Emerging evidence has highlighted the clinical utility of the body roundness index (BRI) and triglyceride‒glucose index (TyG) because of their superior sensitivity in evaluating obesity-related metabolic risk and insulin resistance (IR) [6, 7]. Recent studies have shown that the BRI and TyG index are associated with cardiovascular disease [8–10]. Moreover, depression, a prevalent mental health disorder, has bidirectional pathophysiological relationships with CMM: depressive states may exacerbate metabolic dysregulation through inflammatory activation and hypothalamic‒pituitary‒adrenal (HPA) axis dysfunction, whereas metabolic abnormalities can reciprocally induce depressive symptoms via neuroendocrine pathways, establishing a vicious pathophysiological cycle [11–14]. Studies indicate that insulin resistance significantly promotes the progression of atherosclerosis—the core pathological basis of cardiovascular diseases—through mechanisms such as inducing oxidative stress, impairing endothelial barrier function, and activating inflammatory responses [15]. Notably, depressive states may further amplify cardiovascular risks: Baune et al. confirmed a clear dose-response relationship between the severity of depressive symptoms and immune activation/HPA axis hyperactivity [16]. Current research limitations persist in isolated examinations of individual biomarkers or psychological factors, lacking comprehensive analysis of their tripartite interactions (BRI, TyG, and depression).

This study employs advanced cross-sectional and longitudinal analyses utilizing the China Health and Retirement Longitudinal Study (CHARLS) database to quantify the independent and combined effects of the BRI, TyG index, and depression on CMM risk. Our findings aim to determine whether metabolic indicators and depression mutually potentiate one another to jointly precipitate CMM and establish an evidence-based framework for early CMM detection and multidisciplinary intervention strategies, bridging critical gaps in current preventive cardiometabolic medicine.

## Methods

### Study population

This study utilized the China Health and Retirement Longitudinal Study (CHARLS) database, a nationwide survey conducted in China that was approved by the Biomedical Ethics Committee of Peking University (Ethics Approval Number: IRB00001052–11015) [17]. Since 2011, data have been collected from residents aged 45 years and above in 150 districts or counties across 28 provinces in China via the probability proportional to size (PPS) sampling method. Follow-up surveys were conducted biennially thereafter until 2020. Detailed information about the CHARLS database can be found on its official website (https://charls.pku.edu.cn/).

**Fig. 1:**
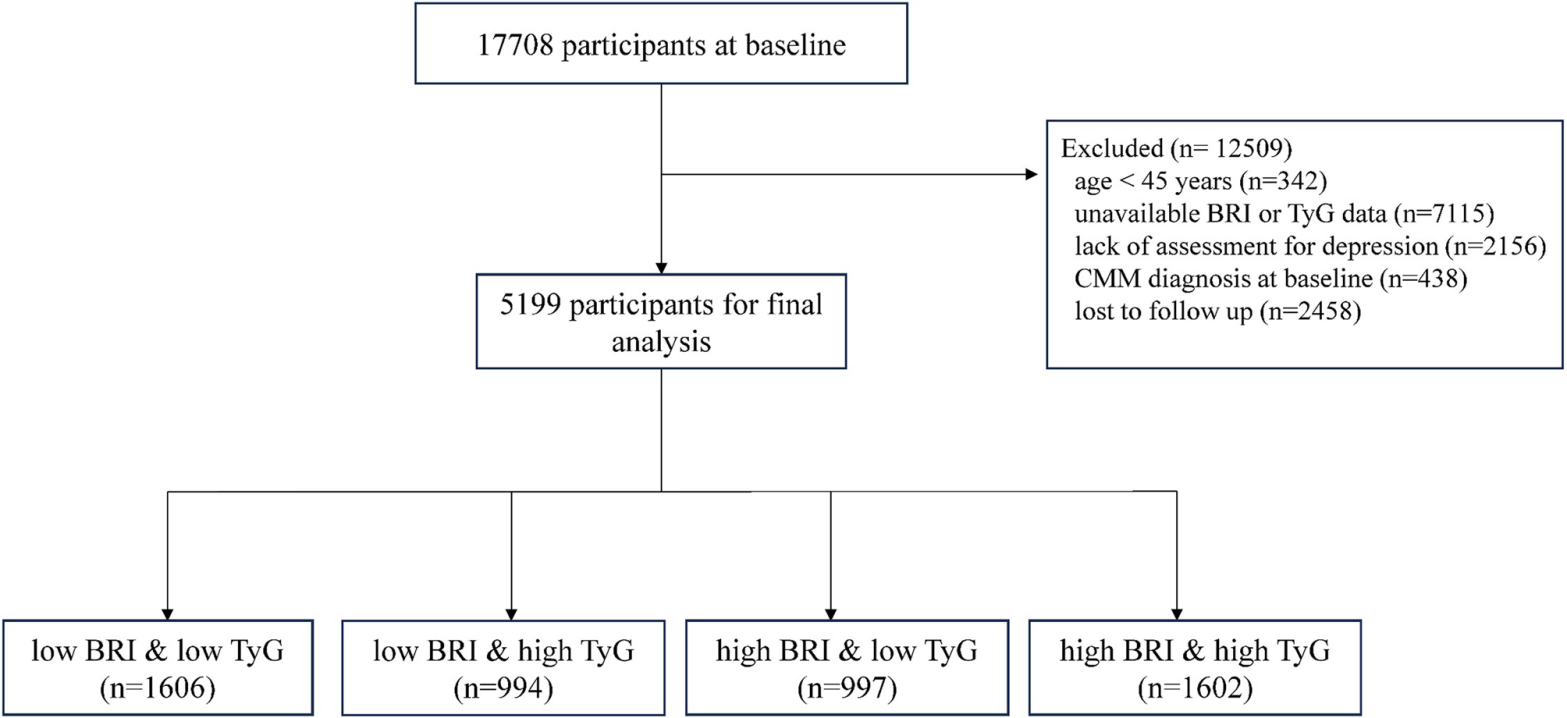
Sample flow chart. *Abbreviations*: *TyG* Triglyceride‒glucose index, *BRI* Body roundness index, *CMM* Cardiometabolic multimorbidity

From the 2011–2018 survey waves, we established a 2011 baseline cohort (*n* = 17,708). Through rigorous quality control protocols, we sequentially excluded: participants aged < 45 years; cases with incomplete anthropometric/biochemical data for TyG/BRI computation; individuals lacking validated depression assessments (CES-D scale); prevalent CMM cases at baseline; and those lost to follow-up. Ultimately, 5,199 individuals were included in the final analysis. The complete participant selection workflow is represented in Fig. 1.

The BRI was calculated via the formula: BRI = 364.2–365.5×$\sqrt{1-\frac{\left({\displaystyle\frac{\mathrm{wc}}2}\times\mathrm\pi\right)2}{0.5\times\mathrm{heigt}2}}$ [6]. The TyG index was calculated via the formula: TyG = ln[TG (mg/dL) × FPG (mg/dL)/2][18]. Depression was assessed using the Center for Epidemiologic Studies Depression Scale (CES-D), whose sensitivity and specificity have been validated in Chinese adults[19, 20]; a score of 10 or higher indicates the presence of depressive symptoms[21, 22].

### Definition of CMM

CMM was defined as the coexistence of two or more of the following conditions: diabetes, stroke, or heart disease[23]. Diabetes was defined as meeting at least one of the following criteria: self-reported diabetes diagnosis; use of diabetes medications; fasting blood glucose ≥126 mg/dL; and a glycated hemoglobin (HbA1c) level ≥6.5%[24]. Heart disease and stroke were diagnosed by a self-reported physician diagnosis.

### Covariates

Baseline covariates included age, sex, household registration, marital status, education level, body mass index (BMI), smoking status, alcohol consumption, hypertension, diabetes, heart disease, C-reactive protein (CRP), high-density lipoprotein cholesterol (HDL-C), low-density lipoprotein cholesterol (LDL-C), total cholesterol (TC), triglycerides (TG), HbA1c, and hemoglobin (Hb).

### Statistical analysis

Baseline characteristics are summarized as the means± standard deviations for continuous variables and frequencies (%) for categorical variables. We first conducted preliminary feature importance analysis using random forest and XGBoost-SHAP to identify key predictors (BRI and TyG). The participants were divided into four groups on the basis of median TyG (8.62) and BRI (4.03) values, with between-group differences assessed via t-tests or χ² tests.

Logistic regression models were employed to compare CMM risk across groups, incorporating multiplicative interaction terms (BRI×TyG) tested via likelihood ratio tests, whereas additive interactions were quantified via relative excess risk due to interaction (RERI) and attributable proportion due to interaction (AP) with 95% confidence intervals (CIs). Further stratification by depression status resulted in eight subgroups. We assessed the BRI × TyG × Depression interaction through stratified regression models and conducted mediation analyses to examine potential mechanistic pathways. To integrate biological and psychological factors, standardized BRI and TyG z scores were combined with depression status to generate a composite cardiometabolic‒psychosocial score via regression coefficients. Predictive performance was compared via ROC analysis (AUC comparisons with DeLong's test) and nonlinear associations were assessed through restricted cubic splines (3 knots). All analyses were performed in R (version 4.4.0), and two-sided *P*- values less than 0.05 indicated statistical significance.

## Results

### Machine learning analysis

**Fig. 2:**
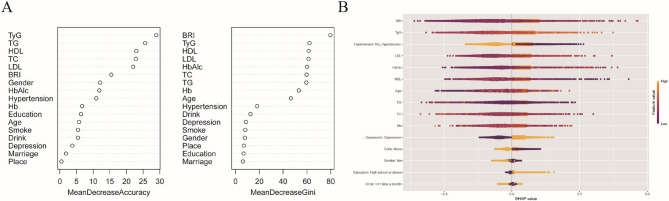
Application of machine learning in feature selection. **A** Feature importance ranking based on Random Forest. Mean decrease accuracy: A larger value indicates a greater contribution of the variable to the predictive accuracy. Mean decrease gini: The reduction in Gini impurity achieved when the variable is used for splitting nodes in the decision trees, reflecting the variable's role in enhancing the purity of sample classifications. **B **SHAP value feature interpretation plot based on XGBoost model. The horizontal position indicates the magnitude of the feature's contribution to the model's prediction (positive on the right, negative on the left), while the vertical axis orders features by their importance. *Abbreviations*: *TyG* Triglyceride‒glucose index, *BRI* Body roundness index, *BMI* Body mass index, *CRP* C‒reactive protein, *Hb* Hemoglobin, *TC* Total cholesterol, *LDL* Low-density lipoprotein cholesterol, *HDL* High‒density lipoprotein cholesterol, *TG* Triglycerides, *HbAlc* Glycated hemoglobin

Preliminary variable importance analysis via machine learning techniques revealed TyG and the BRI as critical predictors of CMM. The random forest model identified these biomarkers as the top contributors on the basis of both mean decrease accuracy and mean decrease gini. XGBoost-SHAP analysis further confirmed their superior predictive capacity, with BRI and TyG demonstrating greater feature importance than traditional measures such as BMI, as visualized in Figure 2.

### Baseline characteristics

This study ultimately included 5,199 participants (2,767 males [53.2%] and 2,432 females [46.8%]) with a mean age of 59.96 years (Table S1). Based on the median values of BRI and TyG, the participants were categorized into four groups: low TyG and low BRI (n=1,606, 30.9%), low TyG and high BRI (n=997, 19.2%), high TyG and low BRI (n=994, 19.1%), and high TyG and high BRI (n=1,602, 30.8%). Notably, the high TyG and high BRI group exhibited significantly higher baseline incidences of hypertension (36.8%) and diabetes (25.8%), coupled with unfavorable lipid profiles characterized by lower HDL and elevated LDL levels. These metabolic abnormalities may contribute to the subsequent high incidence of CMM (11.1%) in this subgroup, suggesting a synergistic association between elevated BRI and TyG indices in driving adverse health outcomes.

### Regression analysis results

As presented in Table 1, elevated levels of both BRI and TyG independently predicted incident CMM, with odds ratios (ORs) exceeding 2.50 (high BRI: OR=2.55, 95% CI=2.01‒3.27; high TyG: OR=2.60, 95% CI=2.04‒3.33) compared to their low-level counterparts. The synergistic exposure group (high BRI and high TyG) demonstrated substantially amplified risk (OR=4.89, 95% CI=3.49‒7.04). Interaction analysis revealed non-significant effect modification between BRI and TyG through both multiplicative (*p* for interaction=0.634) and additive approaches (RERI=1.18, 95% CI=-0.16‒16.50; AP=0.24, 95% CI=-0.02‒0.72). Similar findings have also been observed in stroke and diabetes, whereas a multiplicative interaction between the BRI and TyG index was identified in heart disease. (Table S2).

**Table 1:** Independent and combined effects of TyG index and BRI on CMM risk

Variables	OR(95% CI)^a^	P for interaction^b^	Interaction measures^c^
Low TyG	Reference	——	——
High TyG	2.60(2.04, 3.33)	——	——
Low BRI	Reference	——	——
High BRI	2.55(2.01, 3.27)	——	——
Low BRI & Low TyG (n=40/1606)	Reference	0.634	RERI: 1.18 (-0.16, 16.50) AP: 0.24 (-0.02, 0.72)
Low BRI & High TyG (n=57/994)	2.38(1.58, 3.62)		
High BRI & Low TyG (n=56/997)	2.33(1.55, 3.54)		
High BRI & High TyG (n=178/1602)	4.89(3.49, 7.04)		

*Abbreviations*: *TyG* Triglyceride‒glucose index, *BRI* Body roundness index, *CMM* Cardiometabolic multimorbidity, *OR* Odds ratio, *RERI* Relative excess risk due to interaction, *AP* Attributable proportion due to interaction

^a^ORs were non adjusted

^b^P for interaction term was evaluated through likelihood tests

^c^RERI and AP were calculated based on the reference group with low BRI &low TyG

Subgroup analyses were conducted to examine the associations between BRI and TyG index with incident CMM across diverse populations. The results demonstrated that elevated BRI and TyG index were associated with increased CMM risk in most subgroups. Furthermore, interaction analyses indicated no significant interactions between these indices and factors such as age or sex (Table 2).

**Table 2:** Subgroup analysis of the association of the BRI and TyG index on CMM risk

	Low TyG & Low BRI	Low BRI & High TyG	High BRI & Low TyG	High BRI & High TyG	P for interaction^a^
Gender					0.704
Female	Reference	2.12(1.14,4.04)	1.94(1.09,3.56)	4.18(2.55,7.29)	
Male	Reference	2.46(1.42,4.33)	2.47(1.30,4.64)	5.13(3.14,8.65)	
Age(years)					0.634
<65	Reference	3.23(1.81,5.99)	4.08(2.31,7.50)	9.04(5.54,15.7)	
≥65	Reference	1.64(0.90,3.00)	0.90(0.45,1.74)	1.62(0.94,2.85)	
Hypertension					0.265
No	Reference	2.59(1.55,4.39)	2.30(1.32,4.02)	4.00(2.51,6.56)	
Yes	Reference	1.63(0.82,3.34)	1.40(0.73,2.79)	2.97(1.71,5.48)	
Smoke					0.373
No	Reference	1.98(1.10,3.61)	2.16(1.27,3.78)	4.74(3.01,7.85)	
Yes	Reference	2.76(1.55,5.05)	2.21(1.08,4.42)	4.39(2.55,7.83)	
Drink					0.763
None	Reference	2.66(1.59,4.57)	1.95(1.15,3.38)	4.30(2.77,6.98)	
<1 time a month	Reference	1.89(0.60,6.13)	2.60(0.74,9.14)	3.53(1.27,10.8)	
≥1 time a month	Reference	1.74(0.72,4.19)	3.08(1.34,7.19)	6.97(3.59,14.7)	

*Abbreviations*: *TyG* Triglyceride‒glucose index, *BRI* Body roundness index, *CMM* Cardiometabolic multimorbidity

Models were adjusted for age, gender, education, place, marriage, smoke and drink

^a^P for interaction term was evaluated through likelihood tests

As visualized in Table 3, the integration of depression with metabolic biomarkers revealed a graduated risk pattern. Compared with the reference group (low TyG and low BRI and no depression), all other combinations showed progressively elevated CMM risk. Most notably, the tripartite exposure group (high TyG and high BRI and depression) demonstrated a 14-fold increased risk (OR=14.2, 95% CI=7.95‒27.6). The adjusted logistic regression model revealed significant three-way multiplicative interactions between BRI, TyG index, and depression (*p* for interaction =0.012), with corresponding additive interactions (RERI=4.02, 95% CI=0.03‒11.82; AP=0.52, 95% CI=0.004‒0.92) indicating potential synergistic effects (Table S3). These findings suggest a novel biological pathway through which depression and metabolic dysregulation may jointly potentiate cardiometabolic risk.

**Table 3:** Combined effects of TyG index, BRI and depression on CMM risk

Variables	OR(95% CI)^a^	P for interaction^b^	Interaction measures^c^
Low TyG & Low BRI & No Depression (n=12/1006)	Reference	0.016	RERI = 5.12(-0.16, 16.50) AP = 0.36(-0.01, 0.71)
Low TyG & Low BRI & Depression (n=28/600)	4.05(2.09 , 8.34)		
Low TyG & High BRI & No Depression (n=31/636)	4.24(2.22 , 8.66)		
High TyG & Low BRI & No Depression (n=34/645)	4.61(2.44 , 9.34)		
High TyG & High BRI & No Depression (n=98/1054)	8.49(4.83 ,16.4)		
Low TyG & High BRI & Depression (n=25/361)	6.16(3.13 , 12.8)		
High TyG & Low BRI & Depression (n=23/349)	5.84(2.93 , 12.3)		
High TyG & High BRI & Depression (n=80/548)	14.2(7.95 , 27.6)		

*Abbreviations*: *TyG* Triglyceride‒glucose index, *BRI* Body roundness index, *CMM* Cardiometabolic multimorbidity, *OR* Odds ratio, *RERI* Relative excess risk due to interaction, *AP* Attributable proportion due to interaction

^a^*ORs *were nonadjusted

^b^*P *for interaction term was evaluated through likelihood tests

^c^*RERI* and AP were calculated based on the reference group with low *BRI* & low *TyG* & no Depression

### Mediation analyses

**Fig. 3:**
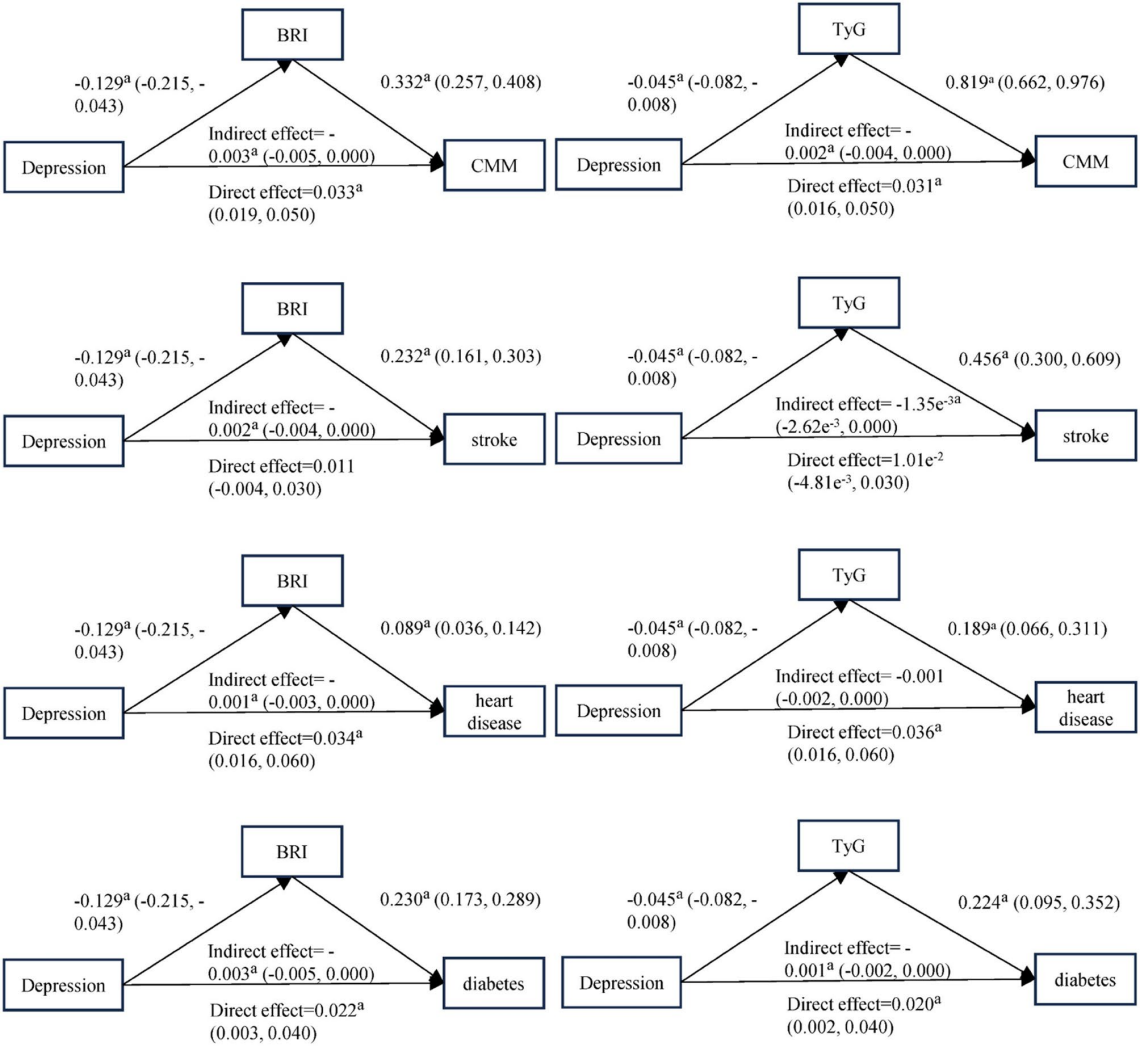
Roadmap of the impact of depression, BRI and TyG on CMM and relative disease.*Abbreviations*:*TyG*Triglyceride‒glucose index, *BRI* Body roundness index, *CMM* Cardiometabolic multimorbidity.^a^*P*<0.05, Models were adjusted by Gender, Age, Marriage,Place and Education

We performed mediation analyses using bootstrap method (Figure3). Results showed that total effect of depression on CMM was around 0.03 (depression - BRI - CMM pathway: 0.033, depression‒TyG‒CMM pathway: 0.031). The mediation effects of BRI and TyG index were -0.003 and -0.002, accounting for -8.82% and -7.87% of the total effect respectively. This indicates depression may reduce CMM risk partly by lowering BRI and TyG index, yet its direct effect still mainly elevates CMM risk. Moreover, the regression coefficients of BRI and TyG index were 0.332 (0.257, 0.408) and 0.819 (0.662, 0.976), showing they promote CMM development directly. Meanwhile, similar mediating effects have also been observed in CMM‒related diseases (Figure 3).

### Predictive performance of integrated risk

**Fig. 4:**
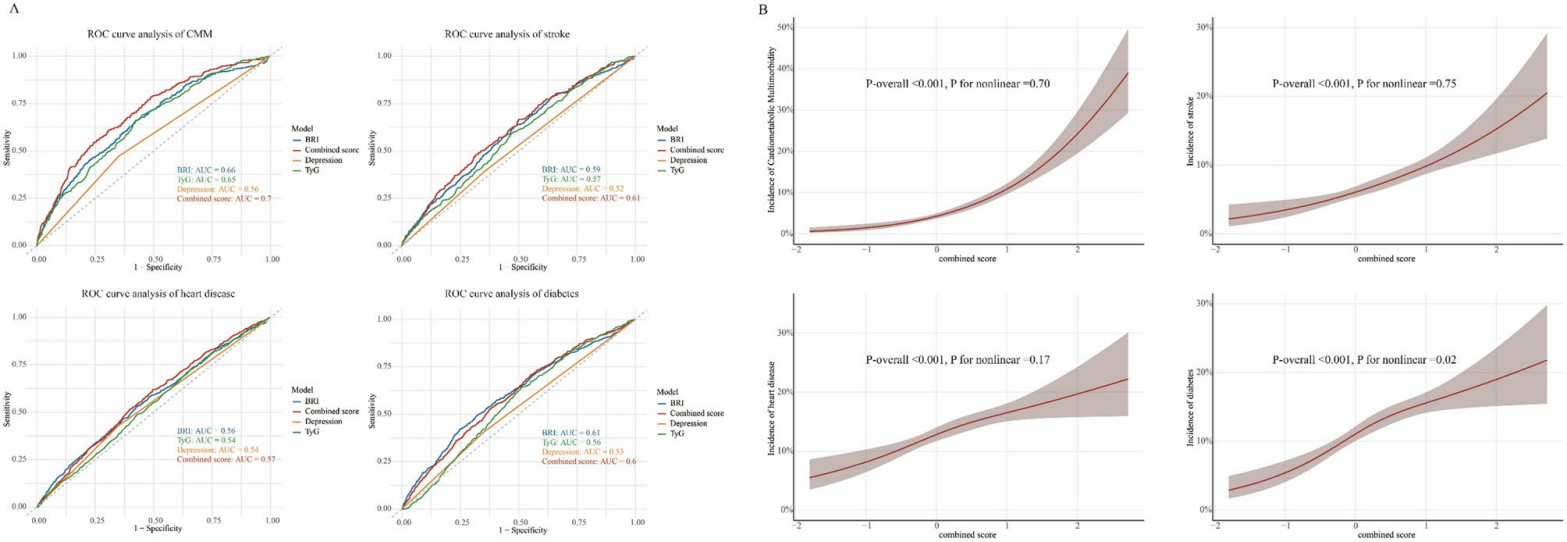
Relationship between CMM and composite score. (A) Predictive Performance of composite score and individual factor for CMM and relative disease. **(B)** Associations between composite score and incidence of CMM and relative disease. *Abbreviations*: *TyG* Triglyceride‒glucose index, *BRI* Body roundness index, *CMM* Cardiometabolic multimorbidity; *AUC* Area under curve

To comprehensively evaluate the synergistic effects of obesity, metabolic dysregulation, and psychological factors, we developed a composite risk score and conducted comparative ROC analysis (Figure 4). The integrated score demonstrated significantly superior discriminative capacity (AUC=0.70, 95% CI=0.66-0.74) compared to individual biomarkers: BRI (AUC=0.66, p<0.001), TyG index (AUC=0.65, p<0.001), and depression (AUC=0.56, p<0.001). However, the predictive power of composite score was not significant in stroke, heart disease and diabetes. On the other hand, RCS analysis with three knots (Figure 4) revealed a dose-response relationship between risk of CMM, stroke, heart disease diabetes and composite score(P-overall <0.001, P for nonlinear =0.70).

## Discussion

This study investigated the independent and synergistic effects of metabolic and psychological indicators on CMM using the CHARLS database. Both BRI and TyG index demonstrated significant positive associations with CMM risk, consistent with existing evidence[25, 26].

BRI, calculated from waist circumference and height, provides superior sensitivity in detecting visceral adiposity compared to BMI[6]. Studies on the relationship between BRI trajectories and cardiovascular disease risk (CVD) have shown a significant dose-response relationship: as BRI increases, CVD risk also rises significantly[8, 27]. This finding is supported by another study in South and Southeast Asian populations, which found that BRI has considerable significance in predicting CVD risk[28]. In this study, individuals with high BRI had a 2.55-fold increased risk of CMM compared to the control group. The mechanism by which elevated BRI increases the incidence of cardiovascular disease may be as follows. Excessive accumulation of visceral fat can induce IR, leading to chronic hyperglycemia[29, 30]. At the same time, excessive visceral fat may lead to dyslipidemia and the release of pro-inflammatory cytokines, further negatively impacting cardiovascular health[31].

TyG, as a validated surrogate marker of IR, reflects lipid metabolism dysregulation and glucose intolerance[32, 33]. Emerging evidence increasingly links the triglyceride-glucose (TyG) index to various cardiovascular diseases, including coronary heart disease (CHD) and stroke[34, 35]. Our study further demonstrates that individuals with a TyG index exceeding 8.62 exhibit a 2.6-fold elevated risk of CMM compared to the reference group. The underlying mechanisms may involve multiple pathways: Firstly, insulin resistance induced by elevated TyG levels promotes macrophage polarization toward a pro-inflammatory phenotype, leading to the release of cytokines such as TNF-α and IL-6[36]. Secondly, insulin resistance may trigger abnormal activation of the renin-angiotensin-aldosterone system, contributing to hypertension development[37]. Furthermore, impaired insulin signaling also dysregulates platelet function, increasing thrombotic tendency[38]. These interconnected pathways collectively exacerbate cardiovascular pathology in individuals with elevated TyG indices.

Emerging evidence supports the depression‒CMM connection[11, 39]. Patients with stroke, heart disease, or diabetes face an elevated risk of depression, potentially mediated by cerebral microcirculatory dysfunction, chronic inflammation, and dysregulation of neurotransmitters such as glutamate[40–43]. Conversely, depression may exacerbate cardiovascular metabolic diseases through mechanisms including endothelial dysfunction, heightened inflammatory responses, platelet activation/aggregation, and behavioral factors such as unhealthy lifestyle choices and reduced medication adherence[43–46]. Our findings further revealing significant interaction effects between depression and metabolic biomarkers (BRI and TyG). These results substantiate the "psychosomatic interaction" hypothesis: depression amplifies metabolic risks through behavioral pathways (sedentary behavior, dietary dysregulation) and biological mechanisms (HPA-axis hyperactivity, chronic inflammation)[14, 47–49], while metabolic abnormalities reciprocally worsen depressive symptoms by impairing quality of life and social functioning, aggravating mitochondrial dysfunction and intestinal flora turbulence, establishing a bidirectional vicious cycle that ultimately elevates cardiovascular disease incidence[50–52].

Depression, BRI and TyG index can elevate the risk of CMM and relative diseases individually or jointly. However, in the unadjusted model, the additive interaction among depression, BRI, and TyG was not statistically significant. Potential reasons for this may include the inherent instability of statistical methods used to assess additive interactions[53]. Additionally, studies on three-way interactions require a large sample size, while the low incidence of CMM (6.4%) in the current study has led to overly wide confidence intervals, resulting in false-negative findings. Notably, depression can also partially reduce this risk by lowering the levels of BRI and TyG index. Potential explanations for this negative association include the comorbidity of depression and anorexia nervosa[54, 55], potentially stemming from shared genetic or environmental factors[56], with gut microbiota also playing a contributory role[57]. Furthermore, depression is more prevalent among individuals with lower socioeconomic status[58, 59]. In China, this often correlates with poorer nutrition and lower body weight[60, 61]. Additionally, the "jolly fat" hypothesis appears more applicable within the Chinese context[62, 63]. Studies consistently demonstrate a lower prevalence of depression among relatively obese individuals in China, a phenomenon which may be attributed to traditional cultural values that view midlife weight gain more positively[64, 65].

This study confirms that individuals with metabolic abnormalities comorbid with depression have a significantly increased risk of CMM, but the interaction between prodromal disease states and depression should not be ignored. A previous Indian study found that patients with prediabetes had higher depression scores and were more prone to comorbid depression[66]. Simultaneously, depression and diabetes progression mutually affect each other; depression can also serve as an independent risk factor for diabetes development[67], which may be closely related to increased HPA axis activity[68]. The association between cardiovascular disease and depression is also complex. Dutch research revealed a significantly higher prevalence of subclinical atherosclerosis in depressed populations[69], and pathological changes such as increased intima-media thickness may be one underlying cause[70]. Wang et al.[71] confirmed the association between clinical/subclinical cardiovascular disease and depression, though the causal relationship remains unclear. Therefore, assessing the metabolic-psychological interaction reveals complex bidirectional associations, and future research should further analyze the causal sequence between the two during the prodromal phase.

Our novel composite score integrating these three domains showed superior predictive performance (AUC=0.70) compared to individual biomarkers. The dose-response relationship between the composite score and CMM incidence underscores the necessity for multidisciplinary interventions targeting concurrent metabolic and mental health management to disrupt this pathogenic cascade. Within relevant hospital departments and community health settings, the combined assessment of a patient's BRI and TyG index alongside depression status (evaluated using the CES-D scale) enables the prediction of CMM risk and facilitates primary prevention. Concurrently, risk stratification allows for the implementation of tiered interventions, ranging from lifestyle modifications for lower-risk individuals to pharmacotherapy combined with cognitive-behavioral therapy for those at higher risk.

This study demonstrates methodological innovation through its integration of novel adiposity indices (BRI) and metabolic markers (TyG) with psychological determinants to elucidate their synergistic effects on CMM, offering clinically actionable insights for integrated care strategies. The nationally representative CHARLS cohort ensures ecological validity for China's aging population, while the logical analytical progression—from biomarker validation to composite risk quantification—enhances result robustness. However, reliance on self-reported cardiovascular endpoints may introduce reporting bias, necessitating future verification with objective clinical measures. Additionally, the lack of comparison between excluded and included participants may lead to selection bias. Furthermore, the use of patients' baseline depression status and biochemical indicators from a biennial survey database risks follow-up bias. Importantly, database limitations precluded the inclusion of key covariates such as socioeconomic status, inflammatory biomarkers (e.g., IL-6), and other mechanistic indicators in our models. This constraint may introduce residual confounding and limit exploration of underlying biological pathways. Therefore, future research should employ shorter follow-up intervals or utilize multiple databases to corroborate the findings. Although the focus on Chinese adults ≥45 years ensures cohort homogeneity, generalizability to younger populations and diverse ethnic groups requires confirmation. Furthermore, the absence of mechanistic investigations highlights critical avenues for subsequent research to unravel biological pathways and optimize personalized interventions.

## Supplementary Information

Supplementary Material 1.

Supplementary Material 2.

Supplementary Material 3.

## Data Availability

Data that support this research can be found in the CHARLS.

## Abbreviations

AP: Attributable proportion due to interaction
AUC: Area under curve
BMI: Body mass index
BRI: Body roundness index
CHARLS: China Health and Retirement Longitudinal Study
CHD: Coronary heart disease
CI: Confidence interval
CMM: Cardiometabolic multimorbidity
CRP: C-reactive protein
CVD: Cardiovascular disease
Hb: Hemoglobin
HbAlc: Glycated hemoglobin
HDL: High‒density lipoprotein cholesterol
IR: Insulin resistance
LDL: Low‒density lipoprotein cholesterol
OR: Odds ratio
RERI: Relative excess risk due to interaction
TC: Total cholesterol
TG: Triglycerides
TyG: Triglyceride-glucose index
